# Supplementary material for: Interactions between interfaces dictate stimuli-responsive emulsion behaviour
Source: Nat Commun. 2023 Oct 23;14:6723. doi: 10.1038/s41467-023-42379-z (PMC10593850; doi:10.1038/s41467-023-42379-z)
Supplement: Supplementary file 1 — Supplementary Information [file 41467_2023_42379_MOESM1_ESM.pdf]

# Supplementary Information: Interactions between interfaces dictate stimuli-responsive emulsion behaviour

*Marcel Rey [1,2,+,*], Jannis Kolker [3,+], James A. Richards [1], Isha Malhotra [3], Thomas S. Glen [1], N. Y. Denise Li [1], Fraser H. J. Laidlaw [1], Damian Renggli [4], Jan Vermant [4], Andrew B. Schofield [1], Syuji Fujii [5,6], Hartmut Löwen [3], Paul S. Clegg [1]*

[1] School of Physics and Astronomy, The University of Edinburgh, Peter Guthrie Tait Road, Edinburgh EH9 3FD, UK.
[2] Department of Physics, University of Gothenburg, SE-41296, Gothenburg, Sweden
[3] Institute for Theoretical Physics II: Soft Matter, Heinrich-Heine University Düsseldorf, D-40225 Düsseldorf, Germany
[4] Department of Materials, ETH Zürich, Vladimir-Prelog-Weg 5, 8093 Zürich, Switzerland
[5] Department of Applied Chemistry, Faculty of Engineering Osaka Institute of Technology, 5-16-1 Omiya, Asahi-ku, Osaka 535-8585, Japan
[6] Nanomaterials Microdevices Research Center, Osaka Institute of Technology, 5-16-1 Omiya, Asahi-ku, Osaka 535-8585, Japan

## Supplementary Discussion

### Interfacial Rheology:

In the following, we will discuss the effect of different interfacial microgel coverages, corresponding to different surface pressures, on the rheological response and the limitations with regards to the measurement setup. We test the following added volumes of 5 mol% microgels (0.15 wt% dispersion): 100 µL added suspension correspond to a surface pressure of 30 mN/m, 50 µL to 24 mN/m, 25 µL to 15 mN/m and 10 µL to 0 mN/m. At 50 µL, interfaces are tested at both a frequency $f$ = 0.2 Hz (Figure 1e,i) and 0.5 Hz, with all other concentrations measured at $f$ = 0.5 Hz alone (Supplementary Figure 3). We report the interfacial storage modulus ($G^{s\prime}$, dark symbols) and loss modulus ($G^{s\prime\prime}$, light symbols) with increasing (filled) and subsequent decreasing strain (open) in Supplementary Figure 2 and Supplementary Figure 3. At 100 µL, through 50 µL and down to 25 µL added microgel suspension, a solid-like jammed interface is found, $G^{s\prime} > G^{s\prime\prime}$, with the linear visco-elastic region extending to a strain of 0.01 (Supplementary Figure 3a,d,g). At 50 µL the elastic response is not found to be dependent of the tested oscillation frequency, cf. Figs 1e and S3d. The stiffness of the 25 µL and 50 µL samples are comparable, $G'_s$ = 0.01 N/m, with the interface at the highest coverage half as stiff. This is consistent with approaching full surface coverage and previous measurements of surface moduli of a similar PNIPAM microgel system.[1,2] With increasing strain amplitude, for all three concentrations the storage modulus drops, and the loss modulus rises, until at $\gamma_0$ = 0.1 to 0.2, the moduli cross, indicating that the interfaces fluidise and yield into a liquid-like state with increasing strain. The behaviour is reversible, cf. open and filled symbols. As the interface is highly elastic before yielding, the ratio of the interfacial stress to the sub-phase drag, the Boussinesq number, is large: Bq = $G^{s\prime}/2\pi f \eta_b l = O(10^3)$, where $\eta_b$ = 2.3 mPas is the sum of the bulk phase viscosities and $l$ = 1 mm the width of the ring. Therefore, we do not correct the data by subtraction of the sub-phase drag, as necessary for lower Bq.[3] With only 10 µL microgel suspension added, no complete microgel monolayer is formed and no significant storage modulus is found (Supplementary Figure 3j). With no measurable response we do not report further results for this concentration.

The set temperature is then increased in a ramp of 0.5 ºC/min to 55 ºC before holding for 30 min; the temperature of the sub-phase is logged every 1 s and the linear visco-elastic properties of the interface are recorded every 30 s at a strain of 0.01, Supplementary Figure 3c,f,i. This allows gradual temperature equilibration to 43 ºC, above the microgel's $T_{VPT}$, Supplementary Figure 3l. The recorded moduli versus measured temperature for 50 µL added are also shown in Figure 1i. At 43 °C, the interface is again characterised using strain sweeps, Fig S3b,e,h. Solid-like interfaces with reversible yielding are again measured with no significant changes in either the moduli or the yield strain above and below $T_{VPT}$ (Figure 1e, cf Supplementary Figure 3a,d,g and S3b,e,h). During the temperature ramp, a slight decrease in both moduli is observed at the longest times. This may be related to mechanical changes of the system as it is repeatedly strained below, but close, to its yielding point. However, this is a small change over long times and this does not indicate that the moduli will cross.

To test for the effect of contact line variations that give rise to false elasticity,[3] a bare water-dodecane interface is measured as a worst-case scenario for surface tension effects (Supplementary Figure 3k). A higher degree elasticity is measured than with 10 µL of microgel, suggesting the measured storage modulus is a result of surface tension effects. The peak modulus $2 \times 10^{-4}$ N/m lies ~ eight-fold below the reported moduli for microgel-covered interfaces. More notably, the peak elastic stress, $G^{s\prime} \times \gamma_0 = 2\ 10^{-6}$ N/m, is two orders of magnitude below the peak elastic stresses for microgel-covered interfaces, confirming that the measured elasticity cannot be ascribed to surface tension artefacts.[3] At high strains, $\gamma_0 > 0.1$, a $G^{s\prime\prime}$ comparable to the sub-monolayer microgel interface is recorded (Supplementary Figure 3j,k). This may originate from sub-phase drag or instrument artefact. The impact of instrument inertia is reduced by measuring at a moderate frequency, 0.2–0.5 Hz, consistent with previous methods on similar systems.[4] Together, the strain sweeps and control tests (Supplementary Figure 3) suggest that the linear visco-elastic moduli reported in Figure1g-j as a function of temperature represent the material response of the interface.

**Emulsion stability under mechanical disturbances:**

To estimate the influence of mechanical disturbances, we compared the behaviour of flocculated and dispersed emulsions under shaking, both at room temperature and at 55 °C. We placed the emulsion vials flat on an incubator shaking plate, which allows us to simultaneously heat and shake. The incubator shaking plate moved in a circle with a diameter of 2 cm and we tested two shaking parameters: 60 rpm and 150 rpm. At 60 rpm, the emulsion was gently shaken back and forth within the vial, while at 150 rpm the flow within the vial was disruptive and chaotic. We compare the emulsions before and after 4 hours of treatment in Supplementary Figure 7.

We observe the following behaviour for dispersed emulsions. Under gentle shaking at 60 rpm the dispersed emulsions remain stable at both room temperature and when heated. The stability of the dispersed emulsion under mechanical disturbance, which brings the droplets into contact, above the $T_{VPT}$ supports our conclusions in the main manuscript, *i.e.* the stability is not an artefact of the quiescent conditions keeping the droplets separated. At higher shaking speed (150 rpm), the dispersed emulsions undergo shear-induced coalescence at both temperatures (clear oil layer on top). Such processes are widely reported, with the stabilisation mechanism being disrupted as the droplets are sheared past one another.[5] Previously, stronger interfaces have been found to resist such shear-induced coalescence, *e.g.,* flocculated *vs* dispersed fumed silica.[6] This suggests that the shear forces at high shaking speeds are sufficient to yield the interfacial monolayer, as in Figure 1. An order of magnitude dimensional analysis estimate from the interfacial yield strength ($\sigma_y \sim 10^{-4}$ Pa.m) and droplet size (10 µm) suggests stresses of ~100 Pa to be sufficient to yield all interfaces consistent with the forces from shaking.

In contrast, for flocculated emulsions shear-induced coalescence is found at both shaking speeds above and below the $T_{VPT}$. We infer that the shear forces acting on the flocculated emulsions are sufficient to break the bridged region of two flocculated droplets in a “zipper-like” manner.[7–9] To summarize, these experiments demonstrated that dispersed emulsions remain stable under mild shaking at 55 °C, but can be broken even at room temperature under high shear.

**Microgel assembly at emulsion interfaces:**

In the following, we discuss the assembly and packing density of microgels (5 mol% crosslinker) stabilizing oil in water emulsions. The interfacial assembly of the microgels in this study have been the subject of prior self-assembly investigations using a Langmuir-Blodgett trough,[10] where the interfacial layer was deposited onto a substrate with the assembly analysed ex situ. At low compression or high area per particle, they assemble into a hexagonal non-close packed arrangement where the microgels are in corona-corona contact.[10–12] Upon further compression, the microgels undergo an isostructural solid-solid phase transition to a hexagonal close packed phase.[10–12] Interestingly, a recent study using small-angle light scattering could not find any evidence of an isostructural phase transition at the liquid interface.[13] Instead, the isostructural phase transition seems to be a result of the microgel’s adhesion to the solid substrate and immersion capillary forces that occur upon drying.[13]

Here, we prepare emulsions with different microgel concentrations and investigate the packing density and assembly of the stabilizing microgels at the droplet interface using cryo-SEM (Supplementary Figure 9). We find a decrease in area per particle (Ap) with increasing microgel concentrations. Further, the microgel assembly at the droplet interface is qualitatively similar compared to our previous study of flat Langmuir monolayers.[10] For low microgel concentrations (0.15 wt%), the microgels are in a hexagonal non-close packed arrangement where they are in corona-corona contact (Supplementary Figure 9a). For intermediate microgel concentrations (0.3 wt%, 0.6 wt%), we find a coexistence of a hexagonal non-close packed and a hexagonal close packed phase (Supplementary Figure 9b,c), which would correspond to the isostructural phase transition. At higher concentrations (1.2 wt%, 1.5 wt%), the assembly is hexagonal close packed (Supplementary Figure 9d,e). Our data suggests that the isostructural phase transition occurs at the liquid droplet interfaces even in the absence of capillary forces upon drying.

With an increase in microgel concentration, the degree of flocculation decreased. In flocculated emulsions (Supplementary Figure 9a-d), we found non-close packed and close packed microgel assemblies. Noteworthily, for 1.2 wt% microgels, most of the emulsion is dispersed but some bridging points in flocculated emulsions can still be observed despite the close packed arrangement. In addition, we find that the microgel assembly in the proximity of bridging points is distorted as the monolayer of the neighbouring droplets interfere with the assembly due to their proximity. The apparent area per particle thus appears higher. Importantly, all investigated dispersed emulsions are characterized by a close packed microgel monolayer (Supplementary Figure 9e), while non-close packed assemblies were only observed in flocculated emulsions (Supplementary Figure 9a-c). These findings qualitatively agree with a study by Destribats et al., who tuned the packing density via the emulsification temperature instead of microgel concentration.[8] Similarly, they report dispersed emulsions for close packed microgel monolayers and flocculated emulsions for non-close packed monolayers.[8]

We should note that controlling the microgel assembly at emulsion interfaces is much more challenging compared to frequently used assemblies in a Langmuir trough. In emulsions, the microgel assembly depends on many different experimental parameters, such as the concentration of microgels in the aqueous

phase (as shown above), their surface to volume ratio, volume concentration adsorption rate, ability to spread at liquid interfaces, size and mobility, swelling ratio and also the emulsification method (*e.g.* shaking, vortex mixing, rotor stator), energy input and oil/water ratio. The detailed influence of some of the mentioned parameters have been elaborated in previous work.[8,9,14,15] The parameters mentioned above are coupled. For example, previous work showed that increasing either microgels crosslinking density or size while keeping a fixed concentration leads to a decrease in packing density at the emulsion interface and an increase in the degree of flocculation.[8,14] In order to qualitatively compare either dispersed or flocculated emulsions (Figure 2), we increase the microgel concentration in the aqueous phase with increasing crosslinking density while keeping the emulsification parameters constant.

**Influence of microgel wetting on emulsion stability:**

Since PNIPAM microgels have a reduced water content in their collapsed state above $T_{VPT}$, it may lead to potential changes in microgel wetting combined with a higher protrusion into the hydrophobic oil phase, even though recent neutron reflectometry experiments could not find any evidence for changes in protrusion with temperature.[16] Our Brownian dynamics simulations allow an exploration of the potential impact of changes in microgel wetting above $T_{VPT}$ to ensure that it is only changes in the swelling of the microgel in the water phase that controls emulsion stability. We increased the width of the attractive potential of the liquid interface to allow the microgel to penetrate more into the oil phase (Figure 4a, grey). Interestingly, we find qualitatively similar trends for increased microgel oil wetting, with repulsive forces for two microgels and attractive forces for one microgel in their collapsed state (Supplementary Figure 10). However, the magnitude of the attractive and repulsive forces is reduced with increasing wetting of the oil phase as the attractive and repulsive forces are primarily induced by the parts exposed to the water phase. Therefore, we conclude that any potential changes in microgel wetting may not be the driving force behind the temperature-induced destabilisation.

**Supplementary Figures:**

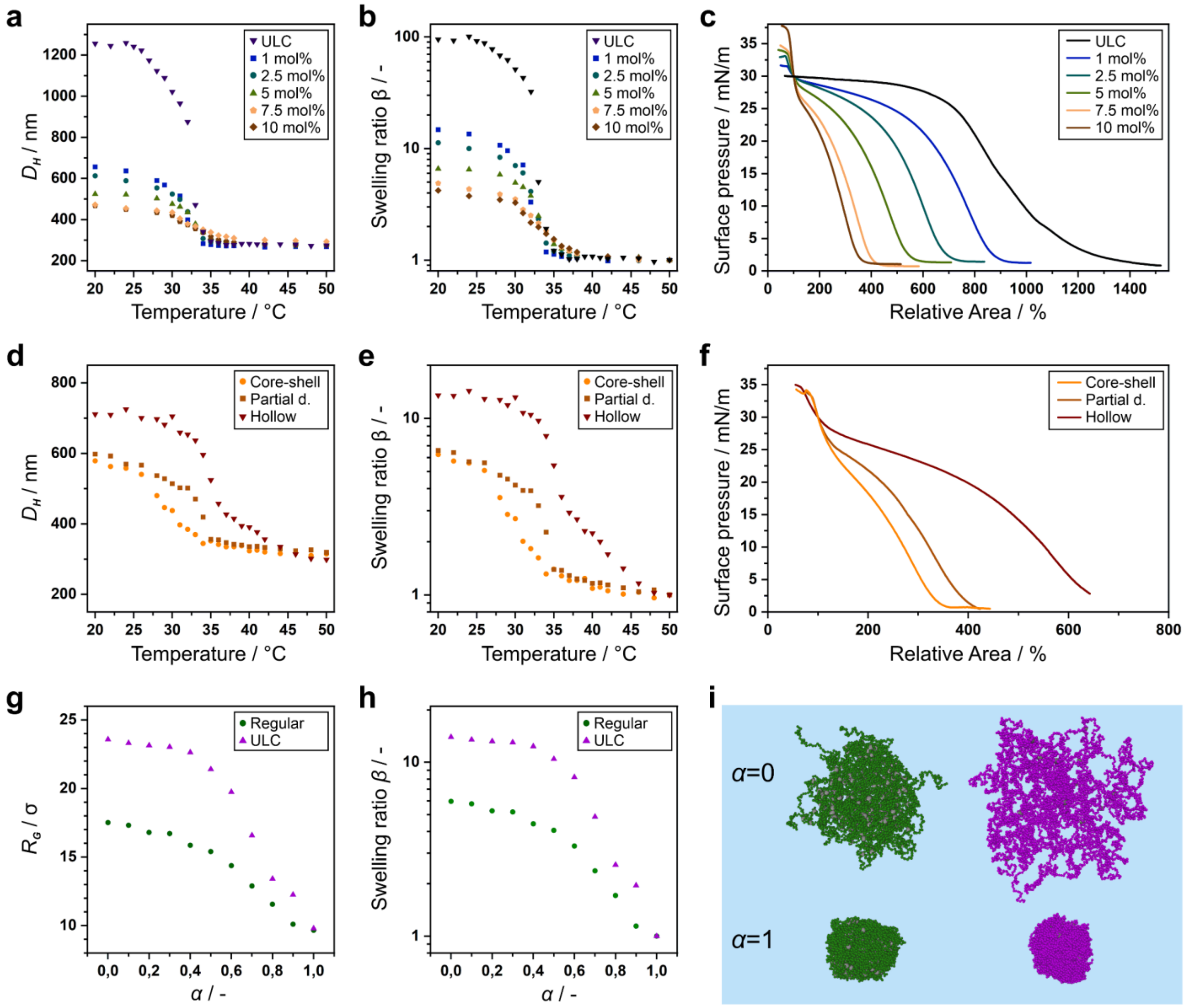


**Supplementary Figure 1:** Bulk and interfacial characterisation of the microgels used in this study. a-c) Series of microgels with increasing crosslinker content from ultra-low crosslinked (ULC) microgels to regular microgels with 1 mol% up to 10 mol% crosslinker. a,b) Hydrodynamic diameter $D_H$ (a) and swelling ratio $\beta$ (b) as a function of temperature. c) Relative area vs surface pressure measured on a Langmuir trough. The area at a surface pressure of 30 mN/m has been defined as 100 %. d-e) Hydrodynamic diameter $D_H$ (d) and swelling ratio $\beta$ (e) as a function of temperature for core-shell microgels, after partially degrading approximately 20 % of the crosslinker from the inner core and hollow microgels. f) Relative area vs surface pressure. g-i) Characterisation of regular and ULC in-silico microgels. g) Radius of gyration ($R_G$) vs $\alpha$. h). Swelling ratio $\beta$ vs $\alpha$. i) Corresponding snapshots in bulk water at $\alpha$=0 and $\alpha$=1.

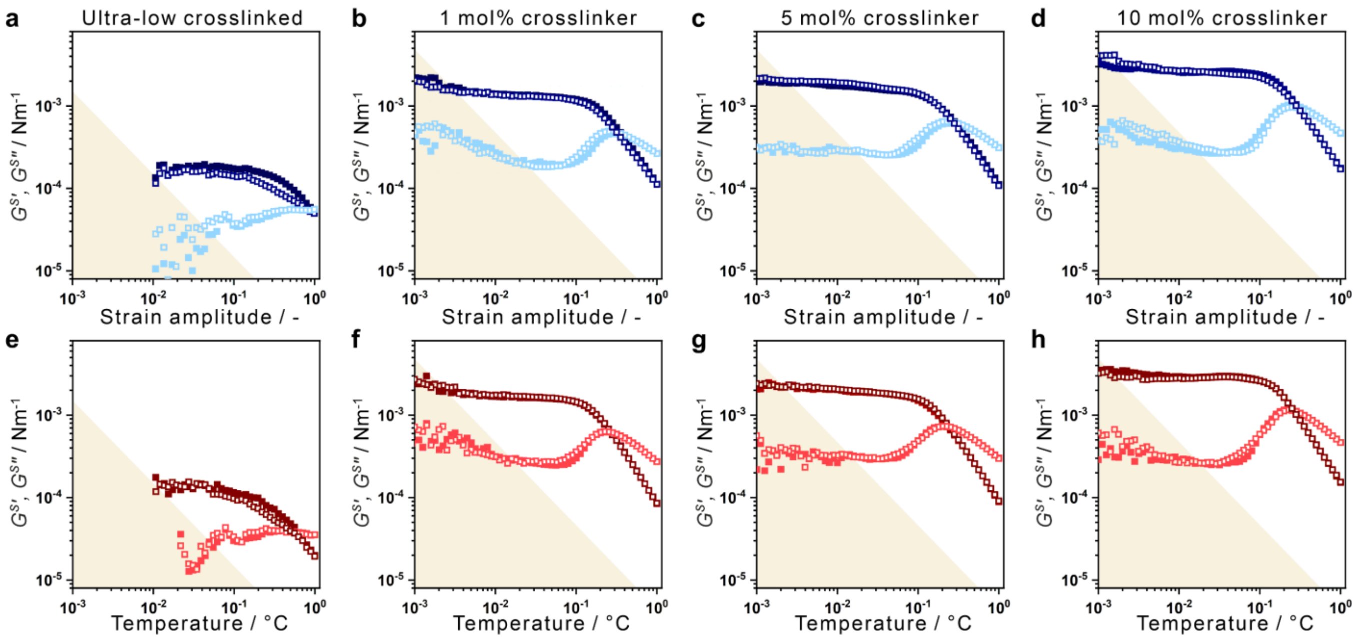


**Supplementary Figure 2:** Interfacial shear rheology oscillatory strain sweeps for microgels with different crosslinking densities below the volume phase transition temperature (a-d, $T<T_{VPT}$, blue symbols) and after increase above (e-h, $T>T_{VPT}$, red symbols). Symbols: interfacial storage modulus, $G^{s\prime}$ (dark), and loss modulus, $G^{s\prime\prime}$ (light), taken with increasing strain amplitude, $\gamma_0$ (filled symbols), followed by decreasing $\gamma_0$ (open symbols). a,e) ULC microgels. b,f) 1 mol% crosslinked microgels. c,g) 5 mol% crosslinked microgels. d,h) 1 mol% crosslinked microgels. Upon decreasing strain, the interface is recoverable for all microgels. Shading: resolution limit.[3]

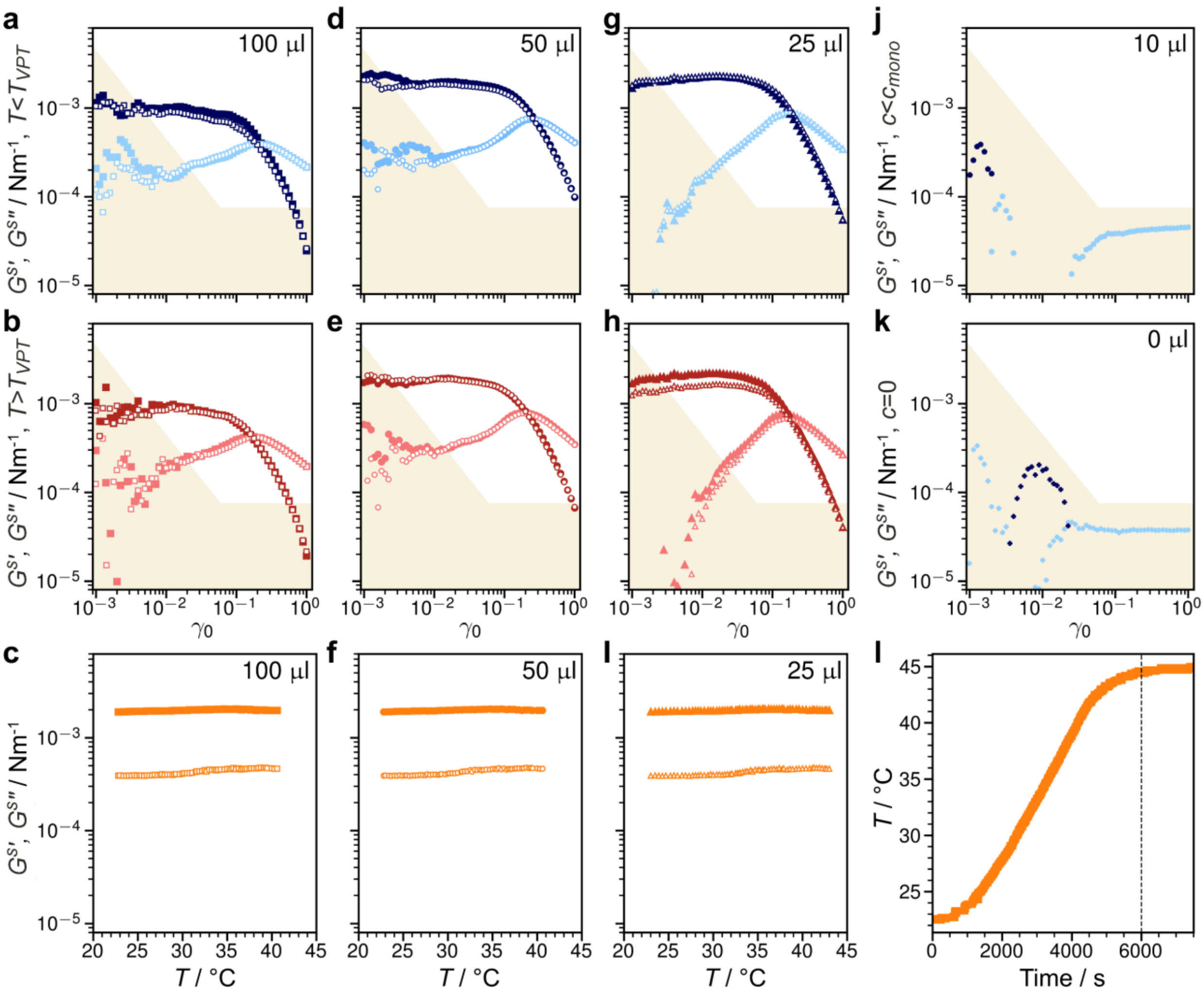


**Supplementary Figure 3:** Interfacial shear rheology oscillatory strain amplitude sweeps with varying surface coverage of regular microgels (5 mol% crosslinker, 0.15 wt%) below the volume phase transition temperature ($T<T_{VPT}$, blue symbols) and after increase above ($T>T_{VPT}$, red symbols). Symbols: interfacial storage modulus, $G^{s\prime}$ (dark), and loss modulus, $G^{s\prime\prime}$ (light), taken with either increasing strain amplitude, $\gamma_0$ (filled symbols), followed by decreasing $\gamma_0$ (open symbols) or increasing temperature at a fixed strain of 0.01. a-c) 100 µL of microgel suspension (~30 mN/m) added to interface (squares). d-f) 50 µL of microgel suspension (~24 mN/m) added to interface (circles). g-i) 25 µL of microgel suspension (~15mN/m) added to the interface (triangle). Low temperature increasing strain sweep only for, j) sub-monolayer coverage using 10 µL of microgel suspension (~0 mN/m) (stars), and k) bare water-dodecane interface. l) Time dependence of temperature during increase, dashed line shows transition from fixed strain monitoring of linear properties to strain sweep. Shading: resolution limit.[3]

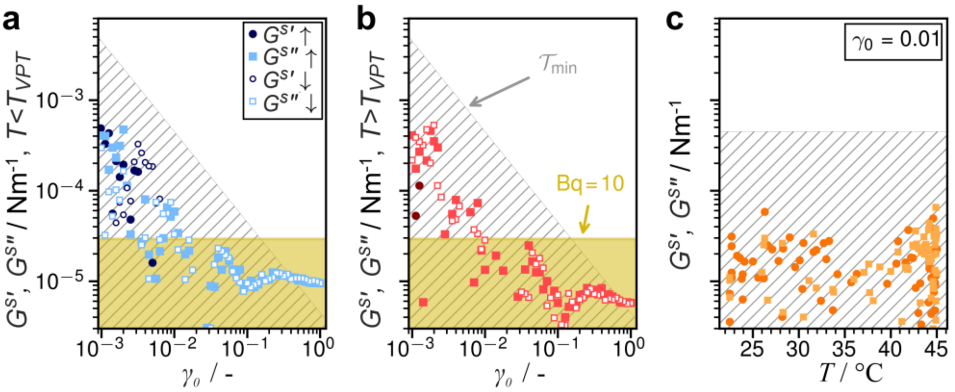


**Supplementary Figure 4:** Interfacial rheology of linear PNIPAM. a) Oscillatory amplitude sweep at $T < T_{VPT}$, interfacial storage modulus, $G^{s\prime}$ (dark), and loss modulus, $G^{s\prime\prime}$ (light), with strain amplitude, $\gamma_0$, for increasing $\gamma_0$ (filled) and decreasing (open). Strain-controlled measurement at $f$ = 0.2 Hz results in indicated limits, instrument torque resolution (grey hatching) and sub-phase drag significance (Bq < 10, yellow shading). b) Equivalent measurement at $T > T_{VPT}$. c) Interfacial moduli during temperature increase at $\gamma_0$ = 0.01; storage, $G^{s\prime}$ (dark), and loss modulus, $G^{s\prime\prime}$ (light). Minimum measurable moduli for experimental parameters, grey hatching. Shading: resolution limit.[3]

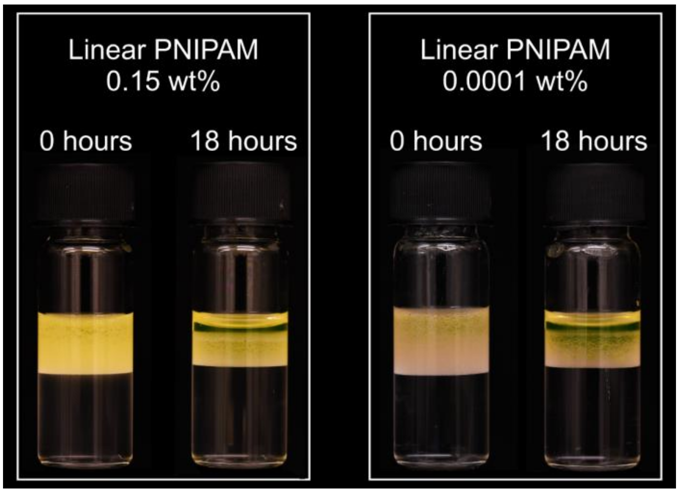


**Supplementary Figure 5:** Dodecane in water emulsions stabilized by linear PNIPAM. These emulsions, independent of the amount of linear PNIPAM used as stabilizer, are not stable at room temperature.

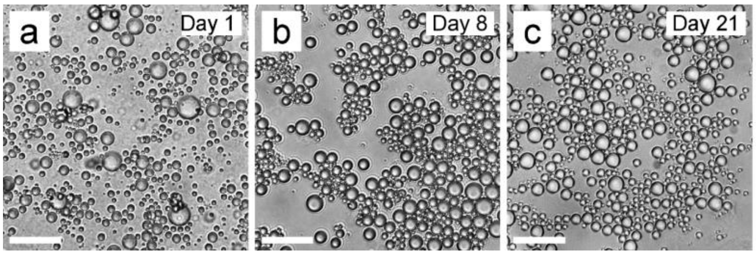


**Supplementary Figure 6:** Evolution of a dispersed emulsion as a function of time stored at 55 °C. Representative microscopy images of the emulsion directly after emulsification (a), after 8 days stored at 55 °C (b) and after 21 days of storage at 55 °C (c). Scale bar: 50 µm.

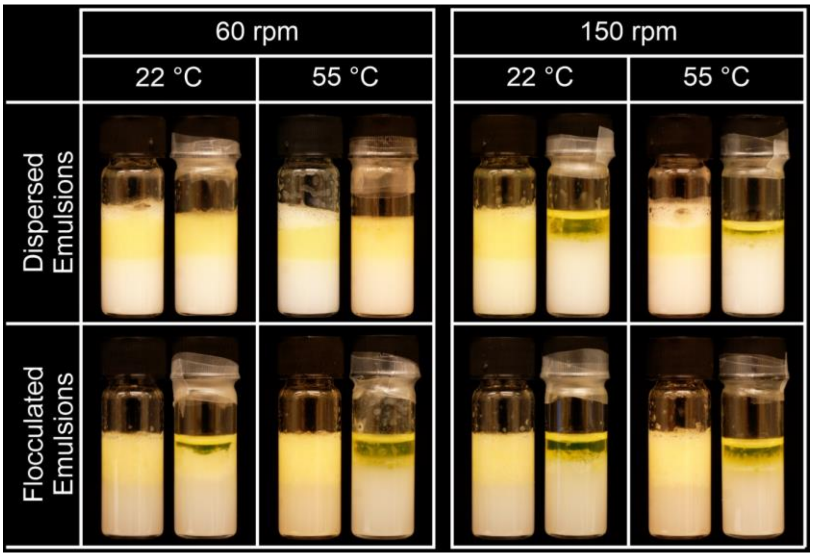


**Supplementary Figure 7:** Simultaneous shaking and heating of dispersed and flocculated emulsions in an incubator. The incubator shaking plate moved in a circle with a diameter of 2 cm at 60 rpm or 150 rpm and the temperature is set to either 22 °C or 55 °C. At 60 rpm, the emulsion was gently shaken back and forth within the vial, while at 150 rpm the flow within the vial was disruptive and chaotic. The vials before (left) and after 4 hours of shaking/heating (right) are shown next to each other for comparison.

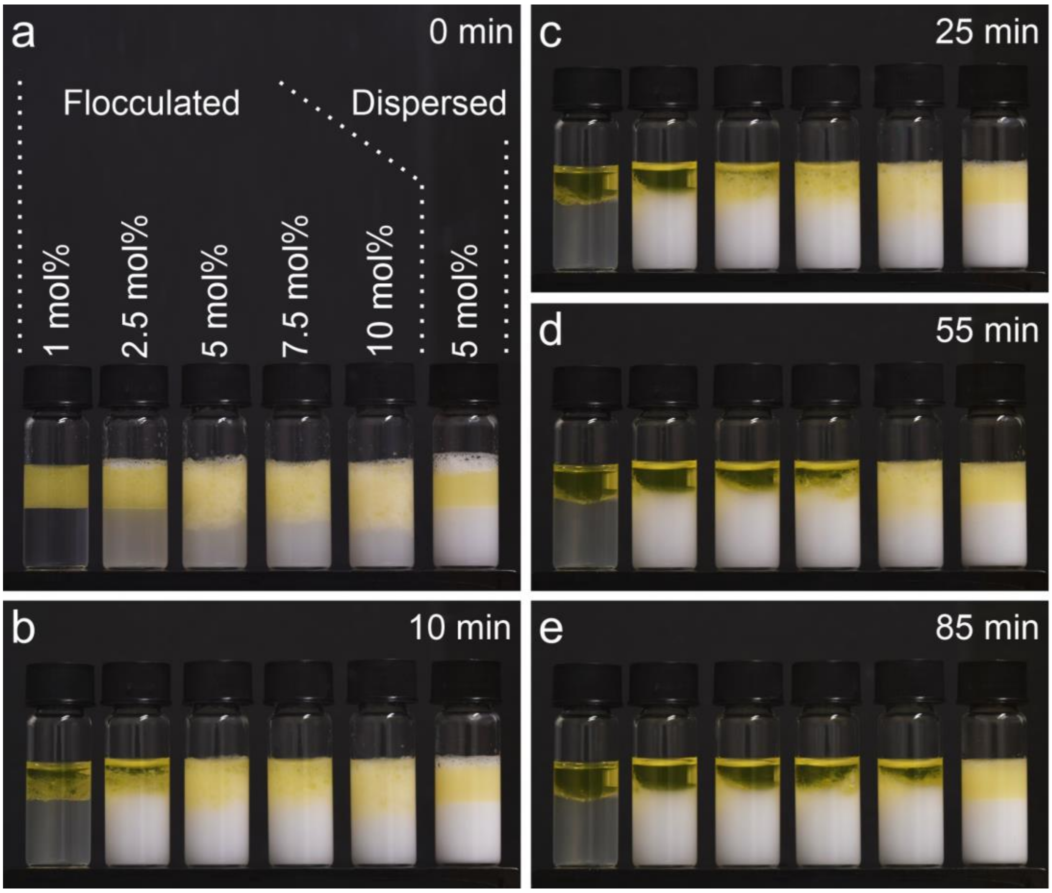


**Supplementary Figure 8:** Time series of dodecane in water emulsions in an incubator at 55 °C. a) Emulsion vials at room temperature. b-e) Emulsion vials during heating to 55 °C after (b) 10 min, (c) 25 min, (d) 55 min and (e) 85 min. Flocculated emulsions stabilized by lower crosslinked microgels break more rapidly compared to the ones stabilized with higher crosslinked microgels, whereas the dispersed emulsion remains stable.

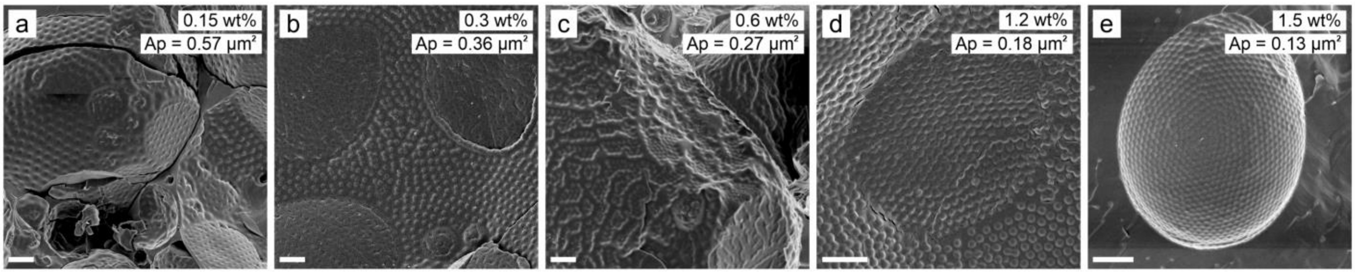


**Supplementary Figure 9:** Cryo-SEM images of dodecane in water emulsions stabilized by 5 mol% PNIPAM microgels. The emulsions are prepared using different concentrations of microgels in the aqueous phase: a) 0.15 wt%, b) 0.3 wt%, c) 0.6 wt%, d) 1.2 wt%, e) 1.5 wt%. With increasing microgel concentrations, we find a decrease in area per particle (Ap). In addition, we observe an iso-structural solid-solid phase transition from a hexagonal non-close packed phase (a) via a phase transition region (b,c) to a close packed phase (d,e), which qualitatively agrees with previous studies on Langmuir monolayers.[10] With increase in microgel concentration, the degree of flocculation decreased. d) For 1.2 wt% microgels, most of the emulsion is dispersed but some bridging points in flocculated emulsions can still be observed despite the close packed arrangement. In addition, we find that the microgel assembly in the proximity of bridging points is distorted as the monolayer of the neighbouring droplet interfere with the assembly due to their proximity. The apparent area per particle thus appears higher. Importantly, all investigated dispersed emulsions are characterized by a close packed microgel monolayer (e), while non-close packed assemblies are only present in flocculated emulsions (a-c). Scale bars: 2 µm.

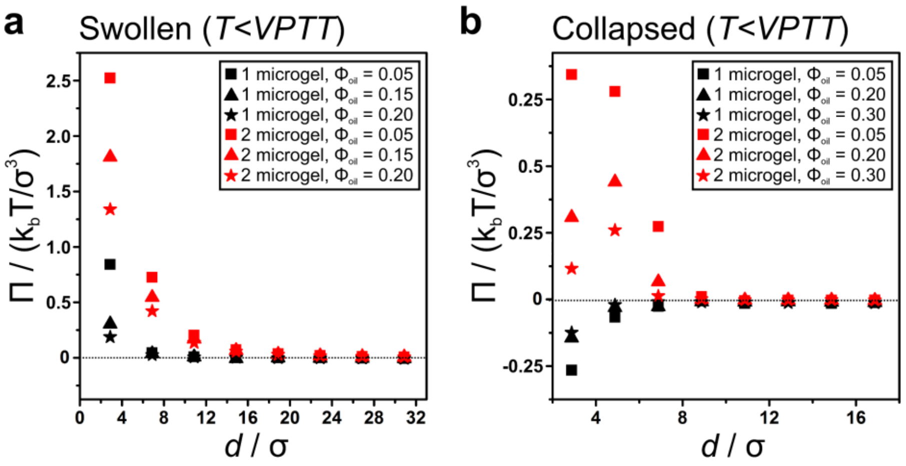


**Supplementary Figure 10:** Monomer-resolved Brownian dynamics simulations of temperature-responsive microgels confined between two planar liquid interfaces in the swollen (a) and in the collapsed (b) state. Osmotic pressure $\Pi$ exhibited by the microgel on the liquid interface as a function of distance between the two liquid interfaces $d$ for one microgel adsorbed to both interfaces (black, representing flocculated emulsions) and two confined microgels each adsorbed to one interface (red, representing dispersed emulsions). Further, the shape of the potential of the oil phase was varied to increase the fraction of beads in the oil phase $\Phi_{oil}$, mimicking a change in microgel wettability. We find qualitatively similar trends for different $\Phi_{oil}$, with repulsive forces for two microgels and attractive forces for one microgel in their collapsed state. For 2 microgels at $\Phi_{oil} = 0.2$ and $\Phi_{oil} = 0.3$ we measure a decrease in the repulsive osmotic pressure $\Pi$ from $d = 6$ to $d = 4$. We attribute this decrease to two effects. First, the possibility of the microgels to partially adsorb to both liquid interfaces in the simulations. Second, attractive bead-bead attractions at high local densities may reduce the osmotic pressure.

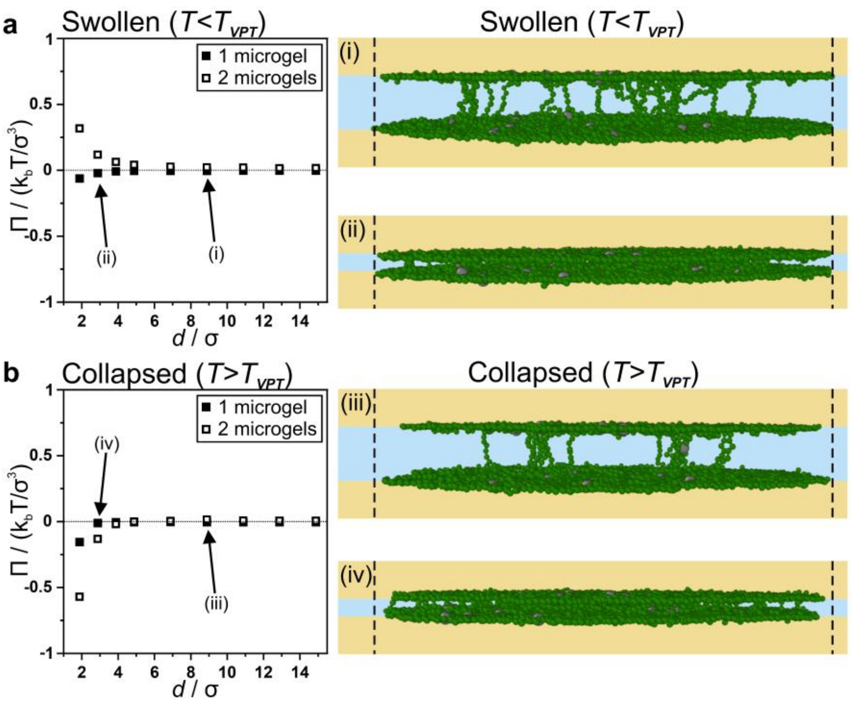


**Supplementary Figure 11:** Monomer-resolved Brownian dynamics simulations of ultra-low crosslinked (ULC) PNIPAM microgels confined between two planar liquid interfaces. a,b) Osmotic pressure $\Pi$ exerted by either one (filled, representing flocculated emulsions) or two (hollow, representing dispersed emulsions) ULC microgels onto the liquid interface as a function of distance $d$ in the swollen (a) and in the collapsed (b) state. A positive $\Pi$ corresponds to a repulsive force of the microgels onto the two liquid interfaces. i-iv) Representative snapshots of one ULC microgel at different compression states in the swollen (i-ii) and collapsed (iii-iv) state.

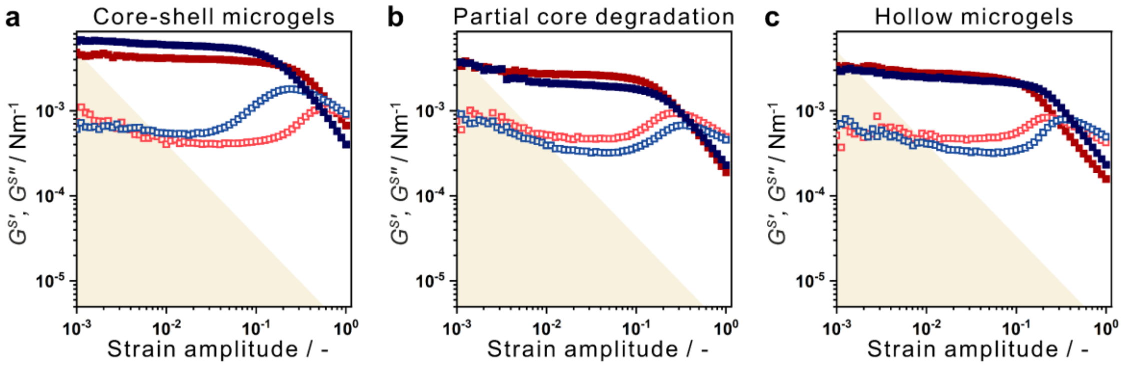


**Supplementary Figure 12:** Interfacial response to changing temperature of thermo-responsive native core-shell microgels, after partial core degradation and complete core degradation (hollow microgels). a-c) Oscillatory strain amplitude sweep for (a) core-shell microgels, (b) core-shell microgels with partial core degradation and (c) hollow microgels at f = 0.2 Hz. Storage ($G^{s\prime}$, filled) and loss ($G^{s\prime\prime}$, open) moduli with strain amplitude, $\gamma_0$, at low temperature, $T < T_{VPT}$ (blue), and high temperature, $T > T_{VPT}$ (red). Shading, resolution for controlled-stress oscillation.[3]

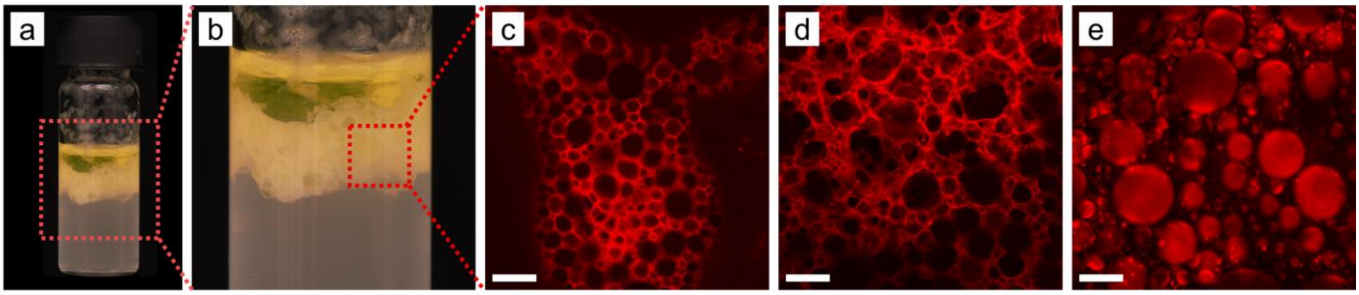


**Supplementary Figure 13:** Confocal microscopy reveals the microstructure of a microgel cluster resulting after de-emulsification of flocculated emulsions. a,b) After de-emulsification, microgels stabilizing flocculated emulsions in part aggregate into a cluster, which sits between the oil and water phase. c,d) Confocal microscopy after transferring parts of the cluster to a cover slip. Note: oil may be drained from the cluster after transfer. Confocal microscopy reveals that the cluster consists of a joint 3D microgel network which persists after droplet coalescence and drainage of the encapsulated oil. We infer that the microgels turn fluorescent due to non-covalent binding of Nile red dye,[17] which we use to visualize the oil phase. e) In addition, some oil droplets are found in the cluster, which likely became trapped during the de-emulsification process, leading to it remaining buoyant between the bulk oil and aqueous phases. The 3D microgel network within the cluster after coalescence indicates that a significant amount of the stabilizing microgels is not able to desorb from the liquid interface and coalescence may instead lead to buckling of the microgel monolayer. Scale bars: 50 µm.

**Supplementary Tables:**

**Supplementary Table 1**: Microgel type, concentration and emulsification method used to obtain the respective emulsions shown in the manuscript.

| Figure | Microgels | Concentration | Emulsification |
|---|---|---|---|
| Figure 2a Dispersed | Linear PNIPAM | 0.15 wt% | Vortex mixing |
| Figure 2a Dispersed | ULC microgels | 0.1 wt% | Vortex mixing |
| Figure 2a Dispersed | 1 mol% microgels | 0.4 wt% | Vortex mixing |
| Figure 2a Dispersed | 2.5 mol% microgels | 1.0 wt% | Vortex mixing |
| Figure 2a Dispersed | 5 mol% microgels | 1.5 wt% | Vortex mixing |
| Figure 2a Dispersed | 7.5 mol% microgels | 2.0 wt% | Vortex mixing |
| Figure 2a Dispersed | 10 mol% microgels | 2.5 wt% | Vortex mixing |
| Figure 2a Flocculated | ULC microgels | 0.1 wt% | Rotor stator |
| Figure 2a Flocculated | 1 mol% microgels | 0.07 wt% | Vortex mixing |
| Figure 2a Flocculated | 2.5 mol% microgels | 0.1 wt% | Vortex mixing |
| Figure 2a Flocculated | 5 mol% microgels | 0.15 wt% | Vortex mixing |
| Figure 2a Flocculated | 7.5 mol% microgels | 0.2 wt% | Vortex mixing |
| Figure 2a Flocculated | 10 mol% microgels | 0.25 wt% | Vortex mixing |
| Figure 2b | ULC microgels | 0.1 wt% | Vortex mixing |
| Figure 2c | 5 mol% microgels | 1.5 wt% | Vortex mixing |
| Figure 2d | 5 mol% microgels | 0.15 wt% | Vortex mixing |
| Figure 3b | 5 mol% microgels | 0.15 wt% | Vortex mixing |
| Figure 3c-e | 5 mol% microgels | 1.5 wt% | Rotor stator |
| Figure 3g-j | 5 mol% microgels | 1.5 wt% | Vortex mixing |
| Figure 3l-o | 5 mol% microgels | 1.5 wt% | Vortex mixing |
| Figure 5a-e | ULC microgels | 0.1 wt% | Vortex mixing |
| Figure 6b-d | Core-shell microgels | 1.2 wt% | Vortex mixing |
| Figure 6g-i | Partially degraded core-shell microgels | 1.0 wt% | Vortex mixing |
| Figure 6l-n | Hollow microgels | 0.4 wt% | Vortex mixing |
| Supplementary Figure 5 | Linear PNIPAM | 0.15 wt% | Vortex mixing |
| Supplementary Figure 5 | Linear PNIPAM | 0.0001 wt% | Vortex mixing |
| Supplementary Figure 6 | 5 mol% microgels | 1.5 wt% | Vortex mixing |
| Supplementary Figure 7 | 5 mol% microgels | 1.5 wt% | Vortex mixing |
| Supplementary Figure 7 | 5 mol% microgels | 0.45 wt% | Vortex mixing |
| Supplementary Figure 8 | 1 mol% microgels | 0.07 wt% | Vortex mixing |
| Supplementary Figure 8 | 2.5 mol% microgels | 0.3 wt% | Vortex mixing |
| Supplementary Figure 8 | 5 mol% microgels | 0.45 wt% | Vortex mixing |
| Supplementary Figure 8 | 7.5 mol% microgels | 0.6 wt% | Vortex mixing |

| | | | |
|---|---|---|---|
| Supplementary Figure 8 | 10 mol% microgels | 0.75 wt% | Vortex mixing |
| Supplementary Figure 8 | 5 mol% microgels | 1.5 wt% | Vortex mixing |
| Supplementary Figure 9a | 5 mol% microgels | 0.15 wt% | Vortex mixing |
| Supplementary Figure 9b | 5 mol% microgels | 0.3 wt% | Vortex mixing |
| Supplementary Figure 9c | 5 mol% microgels | 0.6 wt% | Vortex mixing |
| Supplementary Figure 9d | 5 mol% microgels | 1.2 wt% | Vortex mixing |
| Supplementary Figure 9e | 5 mol% microgels | 1.5 wt% | Vortex mixing |
| Supplementary Figure 13 | 5 mol% microgels | 0.45 wt% | Vortex mixing |

**Supplementary References:**


1. Rey, M. *et al.* Isostructural solid–solid phase transition in monolayers of soft core–shell particles at fluid interfaces: structure and mechanics. *Soft Matter* **12**, 3545–3557 (2016).
2. Tatry, M. C., Laurichesse, E., Vermant, J., Ravaine, V. & Schmitt, V. Interfacial rheology of model water–air microgels laden interfaces: Effect of cross-linking. *J Colloid Interface Sci* **629**, 288–299 (2023).
3. Renggli, D., Alicke, A., Ewoldt, R. H. & Vermant, J. Operating windows for oscillatory interfacial shear rheology. *J Rheol (N Y N Y)* **64**, 141–160 (2020).
4. Brugger, B., Vermant, J. & Richtering, W. Interfacial layers of stimuli-responsive poly-(N-isopropylacrylamide-co-methacrylicacid) (PNIPAM-co-MAA) microgels characterized by interfacial rheology and compression isotherms. *Physical Chemistry Chemical Physics* **12**, 14573 (2010).
5. Yeung, A., Moran, K., Masliyah, J. & Czarnecki, J. Shear-induced coalescence of emulsified oil drops. *J Colloid Interface Sci* **265**, 439–443 (2003).
6. Whitby, C. P., Fischer, F. E., Fornasiero, D. & Ralston, J. Shear-induced coalescence of oil-in-water Pickering emulsions. *J Colloid Interface Sci* **361**, 170–177 (2011).
7. French, D. J., Taylor, P., Fowler, J. & Clegg, P. S. Journal of Colloid and Interface Science Making and breaking bridges in a Pickering emulsion. *J Colloid Interface Sci* **441**, 30–38 (2015).
8. Destribats, M. *et al.* Origin and control of adhesion between emulsion drops stabilized by thermally sensitive soft colloidal particles. *Langmuir* **28**, 3744–3755 (2012).
9. Destribats, M. *et al.* Soft microgels as Pickering emulsion stabilisers: Role of particle deformability. *Soft Matter* **7**, 7689–7698 (2011).
10. Rey, M., Hou, X., Tang, J. S. J. & Vogel, N. Interfacial arrangement and phase transitions of PNiPAm microgels with different crosslinking densities. *Soft Matter* **13**, 8717–8727 (2017).
11. Rey, M. *et al.* Isostructural solid–solid phase transition in monolayers of soft core–shell particles at fluid interfaces: structure and mechanics. *Soft Matter* **12**, 3545–3557 (2016).
12. Rey, M., Fernandez-Rodriguez, M. A., Karg, M., Isa, L. & Vogel, N. Poly- N-isopropylacrylamide Nanogels and Microgels at Fluid Interfaces. *Acc Chem Res* (2020) doi:10.1021/acs.accounts.9b00528.
13. Kuk, K. *et al.* Compression of colloidal monolayers at liquid interfaces: in situ vs. ex situ investigation. *Soft Matter* **19**, 175–188 (2022).
14. Destribats, M. *et al.* Impact of pNIPAM microgel size on its ability to stabilize pickering emulsions. *Langmuir* **30**, 1768–1777 (2014).

15. Destribats, M. *et al.* Pickering emulsions stabilized by soft microgels: Influence of the emulsification process on particle interfacial organization and emulsion properties. *Langmuir* **29**, 12367–12374 (2013).
16. Bochenek, S. *et al.* In-situ study of the impact of temperature and architecture on the interfacial structure of microgels. *Nat Commun* **13**, 3744 (2022).
17. Purohit, A., Centeno, S. P., Wypysek, S. K., Richtering, W. & Wöll, D. Microgel paint-nanoscopic polarity imaging of adaptive microgels without covalent labelling. *Chem Sci* **10**, 10336–10342 (2019).
