# Supplementary material for: Interactions between interfaces dictate stimuli-responsive emulsion behaviour
Source: Nat Commun. 2023 Oct 23;14:6723. doi: 10.1038/s41467-023-42379-z (PMC10593850; doi:10.1038/s41467-023-42379-z)
Supplement: Supplementary file 3 — Description of Additional Supplementary Files [file 41467_2023_42379_MOESM3_ESM.pdf]

Description of Additional Supplementary Files

File Name: Supplementary Movie 1
Description: Accelerated microscopy movie of the behaviour of a dispersed emulsion (ultra-low crosslinked) upon increasing temperature from 28 °C to 53 °C. The movie was accelerated by a factor 80. Area: 238 x 238 μm^2.

File Name: Supplementary Movie 2
Description: Description: Accelerated microscopy movie of the behaviour of a dispersed emulsion (5 mol% crosslinker) upon increasing temperature from 30 °C to 80 °C. The movie was accelerated by a factor 80. Area: 238 x 238 μm^2 .

File Name: Supplementary Movie 3
Description: Accelerated microscopy movie of the behaviour of a flocculated emulsion (5 mol% crosslinker) upon increasing temperature from 28 °C to 44 °C. The movie was accelerated by a factor 80. Area: 238 x 238 μm^2
